# Isolation and Characterization of Urinary Extracellular Vesicles from Healthy Donors and Patients with Castration-Resistant Prostate Cancer

**DOI:** 10.3390/ijms23137134

**Published:** 2022-06-27

**Authors:** Haneul Lee, Su Jin Kang, Jimin Lee, Kyong Hwa Park, Won Jong Rhee

**Affiliations:** 1 Department of Bioengineering and Nano-Bioengineering, Incheon National University, Incheon 22012, Korea; lskyy0828@gmail.com (H.L.); sjkang2525@gmail.com (S.J.K.); 2 Division of Oncology/Hematology, Department of Internal Medicine, Korea University College of Medicine, Seoul 02841, Korea; dlwlals1997@nate.com; 3 Division of Bioengineering, Incheon National University, Incheon 22012, Korea; 4 Research Center for Bio Materials & Process Development, Incheon National University, Incheon 22012, Korea

**Keywords:** liquid biopsy, extracellular vesicle, prognosis, urine, castration-resistant prostate cancer

## Abstract

Prostate cancer (PCa) is the most commonly diagnosed malignancy among men in developed countries. The five-year survival rate for men diagnosed with early-stage PCa is approximately 100%, while it is less than 30% for castration-resistant PCa (CRPC). Currently, the detection of prostate-specific antigens as biomarkers for the prognosis of CRPC is criticized because of its low accuracy, high invasiveness, and high false-positive rate. Therefore, it is important to identify new biomarkers for prediction of CRPC progression. Extracellular vesicles (EVs) derived from tumors have been highlighted as potential markers for cancer diagnosis and prognosis. Specifically, urinary EVs directly reflect changes in the pathophysiological conditions of the urogenital system because it is exposed to prostatic secretions. Thus, detecting biomarkers in urinary EVs provides a promising approach for performing an accurate and non-invasive liquid biopsy for CPRC. In this study, we effectively isolated urinary EVs with low protein impurities using size-exclusion chromatography combined with ultrafiltration. After EV isolation and characterization, we evaluated the miRNAs in urinary EVs from healthy donors and patients with CRPC. The results indicated that miRNAs (miR-21-5p, miR-574-3p, and miR-6880-5p) could be used as potential biomarkers for the prognosis of CRPC. This analysis of urinary EVs contributes to the fast and convenient prognosis of diseases, including CRPC, in the clinical setting.

## 1. Introduction

Prostate cancer (PCa) is the most commonly diagnosed malignancy among men in developed countries and the third leading cause of cancer-related mortality [1]. Early-stage PCa usually develops in an androgen-dependent manner and is treated using androgen deprivation therapy or surgical castration. However, in most patients with advanced PCa, the disease progresses to castration-resistant PCa (CRPC) [2]. In the United States, the average five-year survival rate for early-stage PCa is 100%, while that for CRPC is less than 30% [3,4]. Currently, the detection of prostate-specific antigen (PSA), a biomarker for the prognosis of CRPC, is criticized for its low accuracy, false-positive rate, and high invasiveness due to infection, trauma, and benign prostatic hyperplasia [5,6]. Biopsy specimens of CRPC are difficult to obtain and might not accurately reflect the disease’s progression due to intratumor heterogeneity in patients with CRPC. Therefore, additional prognostic biomarkers are required for the specific and accurate prediction of cancer progression [7]. To accommodate this, it is necessary to develop a novel liquid biopsy method for CRPC detection using human body fluids.

Urine, after blood, is the second most conventionally used biofluid for disease diagnosis, and it presents the opportunity for an accurate, non-invasive liquid biopsy. Urine has enormous potential by providing biomarkers, such as proteins, mRNAs, miRNAs, and extracellular vesicles [8,9]. Biomarkers in extracellular vesicles (EVs) have been extensively studied as novel sources for liquid biopsy [10,11,12,13]. EVs, which are produced from their parental cells, serve as central mediators of cell-to-cell communication. They are nano-sized materials encompassed by lipid bilayers, and multiple components, including proteins, peptides, lipids, carbohydrates, DNAs, and RNAs, are embedded inside them or on their surface [14]. EVs can be directly transferred to their parental or neighboring cells. In addition, EVs can enter the circulatory system and travel through body fluids, including blood and urine [15,16]. This allows the transfer of biomolecules to other cells or tissues, where they subsequently participate in regulating the pathogenesis of various diseases, including cancer [17,18,19,20,21]. Specifically, urinary EVs directly reflect changes in urine in the pathophysiological status of the urogenital system because it is exposed to prostatic secretions [22]. Thus, the detection of biomarkers in urinary EVs provides a promising avenue for achieving accurate and non-invasive liquid biopsies for PCa.

To achieve this, however, high levels of protein impurities in the urine can interfere with biomarker detection by urinary EVs, resulting in false-positive signals [23]. It is necessary to develop a standard method for EV isolation from human urine to ensure high yield and purity. Various methods have been applied to isolate EVs from urine, including filtration, precipitation, hydrostatic dialysis, and size-exclusion chromatography (SEC) combined with ultrafiltration [16]. Among them, EV precipitation can be performed by adding polyethylene glycol (PEG) to urine, and EVs can be recovered via regular centrifugation [24]. Thus, the method facilitates the isolation of EVs from a large number of samples. Despite this advantage, this method has been criticized for its considerably low purity and the fact that it can precipitate many other components, including proteins, along with EVs [25]. Because it was reported that SEC effectively reduced impurities, such as albumin and other proteins in the sample [16], this method is more suitable than PEG-based precipitation for accurate prediction of CRPC progression using EVs from urine. However, because the concentration of EVs in urine is lower than that in serum, a higher volume of urine is needed to isolate EVs. Thus, EV isolation using SEC requires a concentration process, including ultrafiltration (UF), to reduce the volume prior to injection.

**Figure 1:**
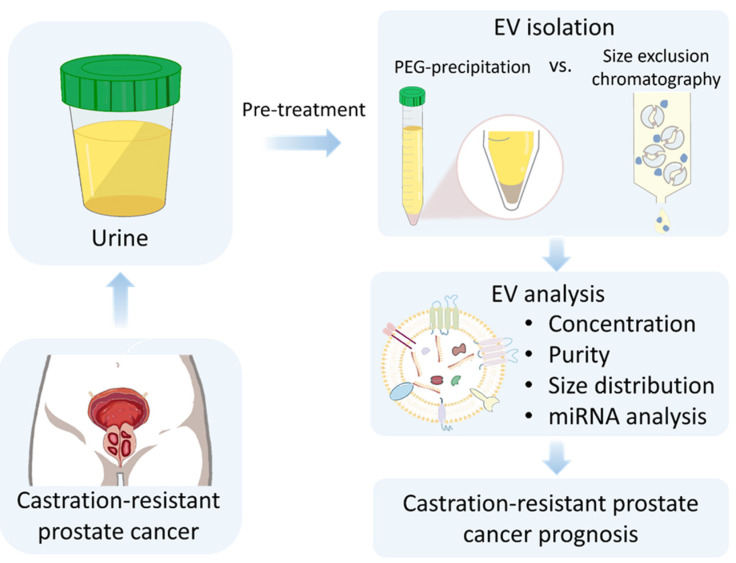
Schematic illustration of extracellular vesicle (EV) isolation using polyethylene glycol (PEG) precipitation or size exclusion chromatography and analysis from the urine of healthy donors and patients with CRPC.

Herein, based on our previous work regarding the EV isolation from the healthy donors [12], we compared the PEG-based precipitation method and SEC combined with UF to separate EVs from healthy donors and patients with CRPC. After EV isolation, the size, concentration, purity, and miRNA profiles of urinary EVs in healthy donors and CRPC patient groups were evaluated (Figure 1). The method and results of this study can contribute to the development of an accurate liquid biopsy method for CRPC using EVs from human urine.

## 2. Materials and Methods

### 2.1. Urine Collection and Pretreatment

Urine samples from patients with CRPC were collected at the Korea University Hospital. The study was approved by the Institutional Review Board of the Korea University School of Medicine and the Incheon National University Institutional Review Board (IRB 7007971-202001-001A). Written informed consent was obtained from all healthy donors and patients with CRPC. Urine samples of 8 healthy donors between the ages of 26 and 53 years were obtained in the morning before smoking and drinking (Table 1). The collected urine samples were stored at −80 °C until further pretreatment. The pretreatment method comprised three steps: low-speed centrifugation (300× *g*, 10 min) to remove cells and medium-speed centrifugation (3000× *g*, 20 min) to remove larger vesicles. After the final centrifugation (17,000× *g*, 20 min), the supernatant was stored at 4 °C while the pellet was incubated with 200 mg/mL dithiothreitol (DTT) (Sigma, St. Louis, MO, USA) at 37 °C for 10 min to release the trapped EVs through the depolymerization of the Tamm-Horsfall proteins [26]. After DTT treatment, the pellet was further centrifuged (17,000× *g*, 10 min), and the supernatants from the centrifugations were mixed and filtered through a 0.22-µm pore size filter.

### 2.2. Urinary EV Isolation Using Ultrafiltration and SEC

Then, 12 mL of pretreated urine were concentrated to 0.5 mL at 5000× *g* for 10 min using an Amicon Ultra-15 Centrifugal Filter Unit (100 kDa MWCO) (Millipore, Bedford, MA, USA). The concentrated sample was added to a qEV size-exclusion column (Izon Science, Christchurch, New Zealand). The fractions (0.5 mL per each faction) were eluted with PBS, and samples were used immediately or stored at −80 °C until further use.

### 2.3. Urinary EV Isolation Using Polyethylene Glycol-Based Precipitation

For polyethylene glycol (PEG)-based precipitation, the modified ExoQuick-TC^TM^ method was used for more efficient isolation of urinary EVs [12]. Briefly, the ExoQuick-TC^TM^ EV precipitation solution (System Biosciences, Palo Alto, CA, USA) was mixed with the pretreated urine at a 3:7 ratio (*v*/*v*), and the mixture was further incubated for 12 h (overnight) at 4 °C, followed by centrifugation at 3000× *g* for 30 min at 4 °C. The pellet containing urinary EVs was dissolved in 1× PBS and stored at −80 °C until further use.

### 2.4. Quantification of EV Particles and Proteins

The number and size of urinary EVs were analyzed via nanoparticle tracking analysis (NTA) using a NanoSight NS300 system (Malvern Panalytical, Malvern, UK). Samples were recorded for three 30-second videos at camera level 14. The protein concentration was quantified using the Bradford assay with a Bio-Rad Protein Assay Reagent (Bio-Rad, Hercules, CA, USA). The Bradford working reagent was used according to the manufacturer’s instructions. Each diluted sample and standard were mixed with the reagent solution, respectively, and incubated at room temperature for 10 min. The absorbance was measured using Varioskan^TM^ Flash Multimode Reader (Thermo Fisher Scientific, Waltham, MA, USA) at 595 nm.

### 2.5. Transmission Electron Microscopy (TEM)

For TEM, the isolated EVs were adsorbed onto copper grids coated only with a thin carbon foil. The sample on the grid was stained with 2% uranyl acetate for 1 min. The grid was washed with distilled water, followed by drying for 15 min. The samples were analyzed using a JEM-1400 Plus electron microscope (JEOL, Akishima, Tokyo, Japan) at the Korea Basic Science Institute in the Republic of Korea.

### 2.6. Western Blot Analysis

For Western blotting, EVs were lysed in 10× RIPA buffer (Millipore, Bedford, MA, USA) supplemented with a protease inhibitor cocktail (Thermo Fisher Scientific, Waltham, MA, USA). Samples were identified under reducing (TSG101) and non-reducing (CD63) conditions, and 10^8^ EV particles were loaded onto (10%) polyacrylamide gels and subjected to sodium dodecyl sulfate-polyacrylamide gel electrophoresis (SDS-PAGE). Separated proteins were transferred to a nitrocellulose membrane with a 0.45-μm pore size (Bio-Rad, Hercules, CA, USA) at 80 V for 2 h. After blocking with 5% skim milk, the membranes were incubated with anti-TSG101 (Abcam, ab83, Cambridge, UK) and anti-CD63 (MBL International Corporation, MEX002-3, Woburn, MA, USA) antibodies overnight at 4 °C, followed by incubation with a horseradish peroxidase-conjugated anti-mouse secondary antibody (Abcam, ab6728, Cambridge, UK). ECL Blotting Reagent (Cytiva, MA, USA) was used for the chemiluminescence reaction. Images were analyzed using a ChemiDoc XRS+ imaging system (Bio-Rad, Hercules, CA, USA).

### 2.7. RNA Extraction and Real-Time Polymerase Chain Reaction (RT-PCR)

RNA was isolated from EVs using a FavorPrep^TM^ Tri-RNA Reagent (Favorgen Biotech Corp., Ping-Tung, Taiwan) according to the manufacturer’s instructions. RNA concentration and purity were analyzed using a NanoDrop^TM^ Lite spectrophotometer (Thermo Fisher Scientific, Waltham, MA, USA). In total, 300 ng of RNA was reverse-transcribed with the Mir-X^TM^ miRNA First-Strand Synthesis Kit (Clontech Laboratories, Palo Alto, CA, USA), which is specific for mature miRNA sequences. RT-PCR was performed using the StepOnePlus Real-Time PCR System (Applied Biosystems, Foster City, CA, USA). To analyze the miRNA expression levels in EVs, U6 small nuclear RNA (snRNA) was used as an internal control for the TB Green Advantage qPCR premix (Clontech Laboratories, Palo Alto, CA, USA). The relative levels of each miRNA were calculated as ΔC_T_ = C_TmiRNA_ − C_TU6_. Comparative quantification was performed using the 2^−ΔCT^ method and is presented as log2 values.

### 2.8. Statistical Analysis

All data were analyzed using GraphPad Prism 7 (GraphPad Software, La Jolla, CA, USA). Data were analyzed using the Mann–Whitney U test and two-way ANOVA, and values were considered statistically significant when *p* < 0.05.

## 3. Results and Discussion

### 3.1. PEG Precipitation-Based Isolation of EVs from Human Urine

**Figure 2:**
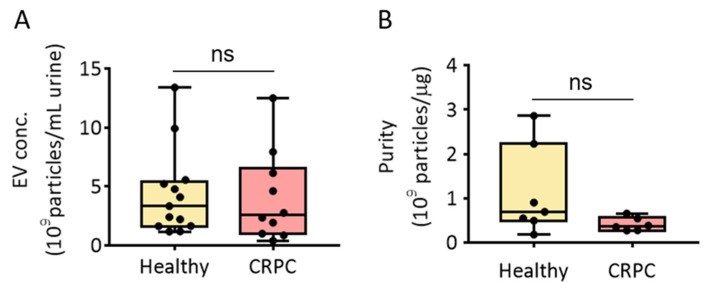
Isolation of urinary EVs via PEG precipitation from healthy donors and patients with CRPC. (**A**) Urinary EV concentration (EV particles/mL urine) from healthy donors and patients with CRPC. (**B**) Urinary EV purity (EV particles/μg protein) from healthy donors and patients with CRPC. All values are presented as the median ± SD (ns: no significance; *n* = 6–13).

The PEG-based precipitation method adopts EV isolation by lowering the solubility of EVs in the solution. We previously reported that modified ExoQuick-TC^TM^ (MEQ) enabled urinary EV isolation from healthy donors [12]. Thus, we first isolated urinary EVs from healthy donors and patients with CRPC using the MEQ method and assessed EV isolation concentration and purity (Figure 2). The results showed that the average concentrations of urinary EVs from healthy donors and patients with CRPC were 3.34 × 10^9^ and 2.56 × 10^9^ particles/mL urine, respectively. However, there was no significant difference in the EV concentration between the two groups (Figure 2A). The purity of isolated EVs was also measured, and no significant difference in urinary EV purity was observed between healthy donors (6.93 × 10^8^ particles/µg protein) and patients with CRPC (3.67 × 10^8^ particles/µg protein) (Figure 2B).

### 3.2. SEC-Based Isolation of EVs from Human Urine

**Figure 3:**
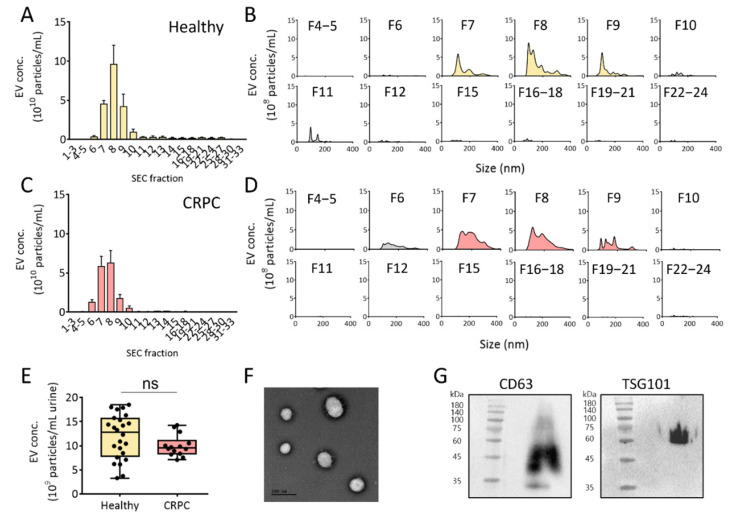
Isolation of urinary EVs via size-exclusion chromatography combined with UF from healthy donors and patients with CRPC. (**A**) Urinary EV concentration (EV particles/mL fraction) from healthy donors. (**B**) Urinary EV size distribution from healthy donors in all size-exclusion chromatography fractions. (**C**) Urinary EV concentration (EV particles/mL fraction) from patients with CRPC. (**D**) Urinary EV size distribution from patients with CRPC in all size-exclusion chromatography fractions. (**E**) Urinary EV concentration (EV particles/mL urine) from healthy donors and patients with CRPC. (**F**) TEM image of isolated urinary EVs. (**G**) Western blot analysis of the EV marker proteins. The number on the left of blot indicates the size marker. All values are presented as the median ± SD (ns: no significance; *n* = 3–24).

Recently, SEC has been applied to separate EVs from other components in a solution, including culture medium and urine. Because neither an additive reagent nor centrifugal force is required for EV isolation, the method allows for the isolation of EVs with higher quality and integrity compared to traditional methods. However, considering the relatively low concentration of EVs in urine, ultrafiltration is a prerequisite for enriching EVs to reduce the sample volume before injection into the SEC column for urinary EV isolation. Based on this, urine samples from healthy donors and patients with CRPC were first concentrated using ultrafiltration, followed by EV isolation using SEC (Figure 3). The concentrations of urinary EVs in each eluted fraction (33 fractions) were measured using NTA. As shown in Figure 3A–D, fractions 7 to 9 from healthy donors and patients with CRPC contained the majority of urinary EVs among the fractions. The average concentrations of EVs isolated from the urine of healthy donors and patients with CRPC were 12.8 × 10^9^ and 9.5 × 10^9^ particles/mL urine, respectively (Figure 3E). However, no significant difference in urinary EV concentration was observed between healthy donors and patients with CRPC. To verify that the isolated EVs maintained typical characteristics of EVs, EVs in fractions 7 to 9 from healthy donors were further analyzed for their shape and the presence of biochemical markers. As shown in Figure 3F, the EVs showed rounded spherical shapes with sizes ranging from 100 to 200 nm, as analyzed using TEM. Western blot analysis of the EV surface marker, CD63, and EV internal marker, TSG101, demonstrated the presence of EVs (Figure 3G).

### 3.3. Comparison of PEG Precipitation and SEC for Urinary EV Isolation

**Figure 4:**
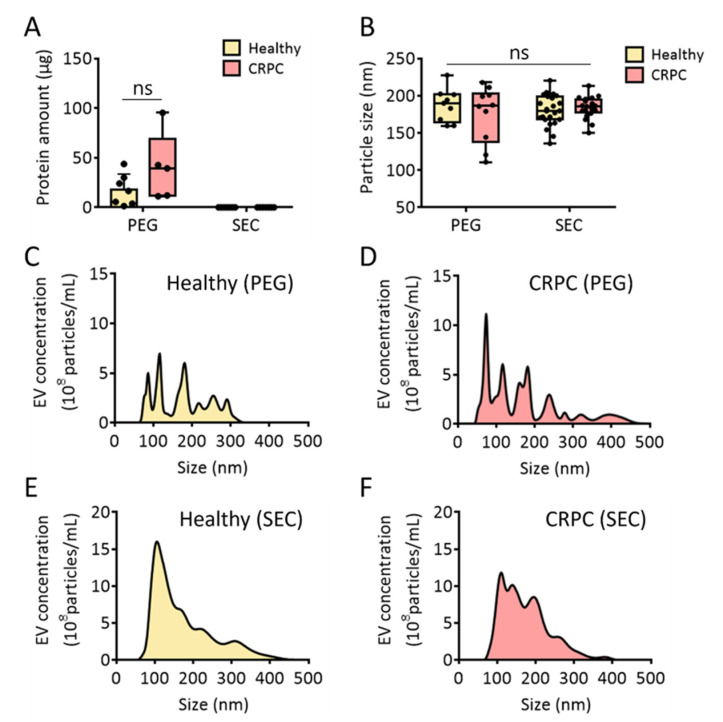
Comparative analysis of urinary EVs isolated via PEG precipitation and size-exclusion chromatography. (**A**) Protein amount (μg) from healthy donors and patients with CRPC. (**B**) Urinary EV particle size (nm) from healthy donors and patients with CRPC. (**C**,**D**) Urinary EV size distribution from healthy donors and patients with CRPC isolated via PEG precipitation. (**E**,**F**) Urinary EV size distribution from healthy donors and patients with CRPC isolated via size-exclusion chromatography combined with UF. All values are presented as the median ± SD (ns: no significance; *n* = 5–24).

To isolate urinary EVs with high yield and purity, the PEG-based EV precipitation method was also tested for EV isolation from both healthy donors and patients with CRPC. In many cases, a high concentration of protein impurities from urine can be co-isolated with urinary EVs. To test this, the same volume of urine was used for comparison between the methods. As shown in Figure 4A, high amounts of protein impurities (17.9 and 40.1 μg in healthy donors and patients with CRPC, respectively) were observed in the urinary EVs isolated through PEG precipitation. Although urinary EVs isolated from CRPC patients contained slightly higher amounts of protein than those isolated from healthy donors, no significant difference was observed. However, when EVs were isolated from urine using SEC, no detectable protein impurities were observed in either healthy donors or patients with CRPC. This clearly demonstrates that SEC ensured urinary EV isolation with high purity.

The sizes of urinary EVs isolated using each method were also compared, and there were no significant differences in the average EV sizes. The average sizes of EVs isolated via PEG precipitation were 187.3 and 175.6 nm for healthy donors and patients with CRPC, respectively. Moreover, the average sizes of isolated urinary EVs using SEC were 179.9 and 184.4 nm for healthy donors and patients with CRPC, respectively. However, no statistically significant difference was observed among the groups, indicating that the first isolation method did not affect the size of isolated particles. Moreover, the results suggested that CRPC did not affect the average size of EVs (Figure 4B). Interestingly, the size distribution profile analyzed through NTA showed that the PEG precipitation method produced multiple major nanoparticle peaks (Figure 4C,D). In contrast, urinary EV isolation via SEC resulted in relatively homogenous peaks, regardless of cancer occurrence (Figure 4E,F). This indicates that PEG precipitation may induce aggregation of EVs with other EVs. Additionally, protein impurities can be associated with EVs or themselves to produce heterogeneous nanoparticles. It was also reported that a large amount of EVs with the size ranging from 50 to 150 nm was obtained by SEC while PEG precipitation produced particles with various sizes [27]. Overall, we concluded that urine concentration using UF followed by EV isolation using SEC helps produce urinary EVs with minimal protein impurities.

### 3.4. Evaluation of Cancer-Related miRNA Levels in Urinary EVs from Healthy Donors and Patients with CRPC

**Figure 5:**
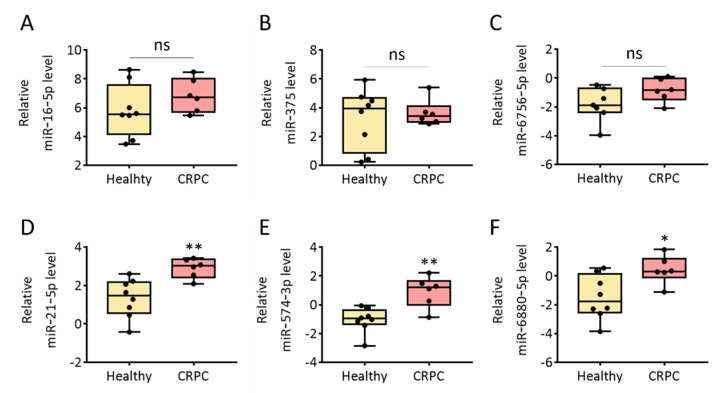
Relative expression levels of cancer-related miRNAs in urinary EVs from healthy donors and patients with CRPC. (**A**) miR-16-5p, (**B**) miR-375, (**C**) miR-6756-5p, (**D**) miR-21-5p, (**E**) miR-574-3p, and (**F**) miR-6880-5p in urinary EVs from healthy donors and patients with CRPC analyzed using RT-PCR and normalized (ΔC_T_ analysis) to expression levels for the U6 snRNA gene and are shown as log2 value. All values are presented as the median ± SD (* *p* < 0.05, ** *p* < 0.01, ns: no significance; *n* = 6–8).

EVs are ideal sources of biomarkers for the diagnosis and prognosis of diseases, including PCa [28]. EVs isolated from human urine can be widely used for liquid biopsies. Some disease-related miRNAs are encapsulated in EVs, secreted into the body fluid, and circulated through the body. To test if the isolated urinary EVs contained specific miRNAs, we chose six miRNAs (miR-16-5p, miR-375, miR-6756-5p, miR-21-5p, miR-574-3p, and miR-6880-5p) that were identified as potential biomarkers for differentiating cancer from healthy donors based on previous studies [29,30,31,32,33]. Real-time PCR analysis of each miRNA in the urinary EVs isolated using SEC from healthy donors and patients with CRPC was performed to assess miRNA levels. The amount of each miRNA was normalized with U6 snRNA level of the same sample described in the ‘Materials and Methods’ section. Because the urinary EV miRNA profiles can vary among individuals of different ages, EV miRNA profiles from healthy donors were compared with different ages. As shown in Figure S1, there were no significant differences in the most of miRNAs except miR-16-5p between two groups of different ages. Further investigation should be undertaken to explore the EV miRNA profiles with ages over 60. First, the relative levels of miR-16-5p in healthy donors and patients with CRPC were 5.55 and 6.73, respectively (Figure 5A). However, there was no statistically significant difference between the two groups (*p* = 0.345). This is consistent with a previous report that miR-16-5p can be used as an internal control for EVs because its level does not vary among humans or across disease states [34,35,36]. In addition, the relative levels of miR-375 and miR-6756-5p were 3.95 and −1.88, respectively, in EVs from healthy donors and 3.40 and −0.86, respectively, in EVs from patients with CRPC (Figure 5B,C). It was reported that the overexpression of miR-375 prevented the secretion of metastasis-suppressive proteins by down-regulation of SEC23 homolog A and Yes-associated Protein 1 [37,38]. Furthermore, overexpression of miR-6756-5p in CRPC cells was reported, miR-6756-5p decreased the expression of IL-24 that has tumor suppressor activities [39,40]. However, there was no significant difference in expression of the miR-375 (*p* = 0.852) and miR-6756-5p (*p* = 0.181) between the two groups.

In contrast, we found significantly higher levels of miR-21-5p, miR-574-3p, and miR-6880-5p in patients with CRPC (Figure 5D–F). The average levels of miR-21-5p were 1.46 and 3.02 in EVs isolated from healthy donors and patients with CRPC, respectively, which was equivalent to a 2.07-fold increase in urinary EVs from patients with CRPC. Moreover, the relative levels of miR-574-3p and miR-6880-5p were −0.97 and −1.76, respectively, in EVs from healthy donors and 1.20 and 0.32, respectively, in EVs from patients with CRPC. Previously, it has been reported as a prognostic biomarker through changes in miR-21-5p according to the advanced prostate cancer patients receiving androgen deprivation therapy [41]. Overexpression of miR-21-5p has been reported to downregulate programmed cell death protein 4, which is a target of androgen receptor signaling and a regulator of PCa cell growth, survival, and castration resistance [42]. In addition, Phosphatase and tensin homolog (PTEN) is a target gene of miR-21-5p, which involves tumor cell growth, metastasis and invasion by downregulating the expression of PTEN [43]. Thus, our results demonstrated that miR-21-5p in urinary EVs can be further investigated as a biomarker for CRPC using a large cohort study. In addition, miR-574-3p was reported to be overexpressed in urinary EV of PCa patients [44], miR-574-3p was involved in the regulation of the Notch signaling pathway, Wnt signaling pathway, apoptosis, DNA damage response, inflammatory response pathway and angiogenesis [45]. Previous studies have not reported the role of miR-6880-5p in PCa. Our findings revealed that miR-6880-5p could be a candidate biomarker for CRPC. Overall, miR-21-5p, miR-574-3p, and miR-6880-5p in human urinary EVs can be further investigated as liquid biopsy biomarkers for CRPC. The method and results investigated in this study offer great opportunities for non-invasive liquid biopsy of CRPC using EVs from urine.

## 4. Conclusions

We showed that EVs could be efficiently isolated through a two-step process, including urine concentration through ultrafiltration and EV isolation via SEC. The method drastically lowered the amount of protein impurities in the isolated urinary EV solutions compared to the PEG precipitation method. The size distribution of EVs from the NTA results confirmed that a relatively homogenous population of EVs was isolated. Analysis of urinary EV miRNAs demonstrated that the method showed promise for applications, such as the non-invasive liquid biopsy of CRPC. Overall, the method allows for a feasible analysis that can be used for the fast and accurate prediction of disease progression, including CRPC.

## Figures and Tables

**Table 1:** Demographic and clinicopathological characteristics of castration-resistant prostate cancer patients recruited to the study.

	Age (years)		Tumor Stage	Gleason Score				PSA (ng/mL)	
	Mean ± SD	Range	T4	6	7	8	9	Mean ± SD	Range
Patients with CRPC (*n* = 6)	76.8 ± 5.9	68–83	6	1	2	2	1	171.1 ± 254.2	0.236–600

## Data Availability

The data presented in this study are available on request from the corresponding author.

## Supplementary Materials

The following supporting information can be downloaded at: https://www.mdpi.com/article/10.3390/ijms23137134/s1.
