# Supplementary material for: Isolation and Characterization of Urinary Extracellular Vesicles from Healthy Donors and Patients with Castration-Resistant Prostate Cancer
Source: Int J Mol Sci. 2022 Jun 27;23(13):7134. doi: 10.3390/ijms23137134 (PMC9266865; doi:10.3390/ijms23137134)
Supplement: Supplementary file 1 [file ijms-23-07134-s001.zip › ijms-1752111-supplementary.pdf]

__Supplementary Information__

# Isolation and characterization of urinary extracellular vesicles from healthy donors and patients with castration-resistant prostate cancer

**Haneul Lee[1], Su Jin Kang[1], Jimin Lee[2], Kyong Hwa Park[2*] and Won Jong Rhee[1,3,4,*]**

1 Department of Bioengineering and Nano-Bioengineering, Incheon National University, Incheon 22012, Republic of Korea.
2 Division of Oncology/Hematology, Department of Internal Medicine, Korea University College of Medicine, Seoul 02841, Republic of Korea
3 Division of Bioengineering, Incheon National University, Incheon 22012, Republic of Korea.
4 Research Center for Bio Materials & Process Development, Incheon National University, 119 Academy-ro, Yeonsu-gu, Incheon 22012, Republic of Korea
* Correspondence: W.J.Rhee, Tel: +82 32 835 8299; Fax: +82 32 835 0763;
Email: wjrhee@inu.ac.kr; K.H.Park, Email: khpark@korea.ac.kr

**Supplementary Information includes:**

Figure S1: Relative expression levels of cancer-related miRNAs in urinary EVs from healthy donors.

## Supplementary figures:

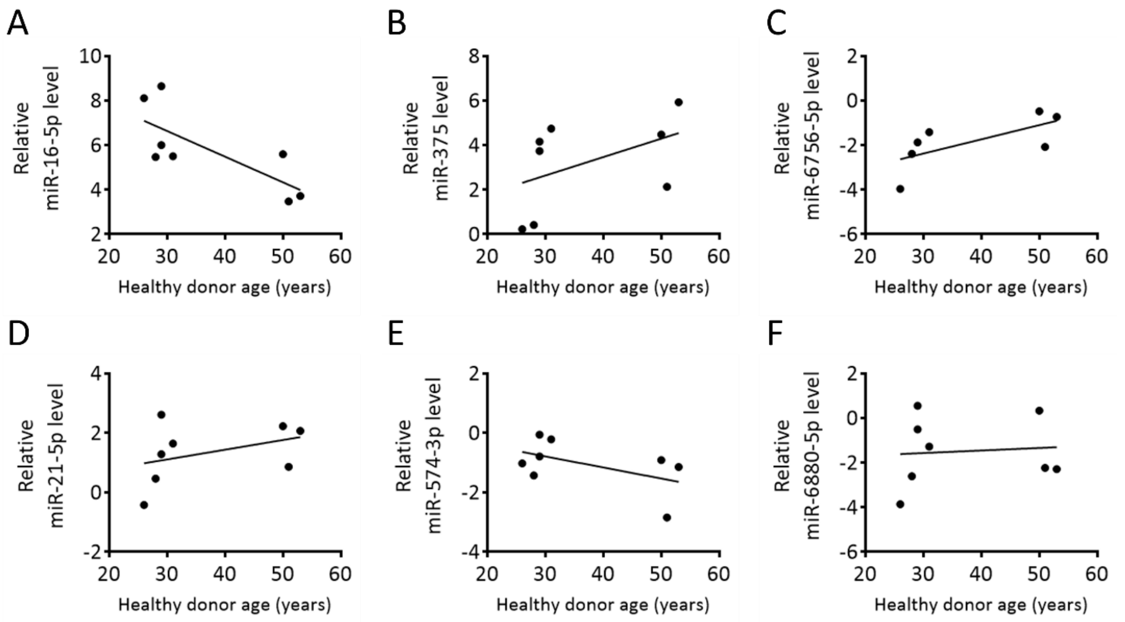


**Figure S1.** Correlations between age and individual miRNA levels in urinary EVs from healthy donors. Correlation of age with (A) miR-16-5p, (B) miR-375, (C) miR-6756-5p, (D) miR-21-5p, (E) miR-574-3p, and (F) miR-6880-5p. Relative miRNA levels in urinary EVs from healthy donors were analyzed using RT-PCR and normalized ($\Delta C_T$ analysis) to expression levels for the U6 snRNA gene and are shown as log2 value. Correlation of age with miRNA expression levels was calculated using Pearson's correlation.
